# Traction-Assisted Endoscopic Submucosal Dissection with Novel Hemostatic Powder Application for a Carbon Nanoparticle-Tattooed Colonic Lesion

**DOI:** 10.1055/a-2933-8288

**Published:** 2026-08-25

**Authors:** Yue Sun, Shishir Karna, Yue Li, Bixiong Zhang

**Affiliations:** 1 Center of EndoscopyGuangdong Provincial People’s Hospital, Guangdong Academy of Medical Sciences, Southern Medical UniversityGuangzhouGuangdongChina

## 

**Video 1** Traction-assisted endoscopic submucosal dissection with novel hemostatic powder application for a carbon nanoparticle-tattooed colonic lesion.


Carbon nanoparticle tattooing is widely used for colorectal lesion localization, but it frequently induces submucosal carbon deposition, obscuring the dissection layer and posing technical difficulties to subsequent endoscopic submucosal dissection (ESD). 1 2 We report a carbon nanoparticle-tattooed colonic lesion successfully managed by traction-assisted ESD and novel hemostatic powder ( Video 1 ).

**Fig. 1:**
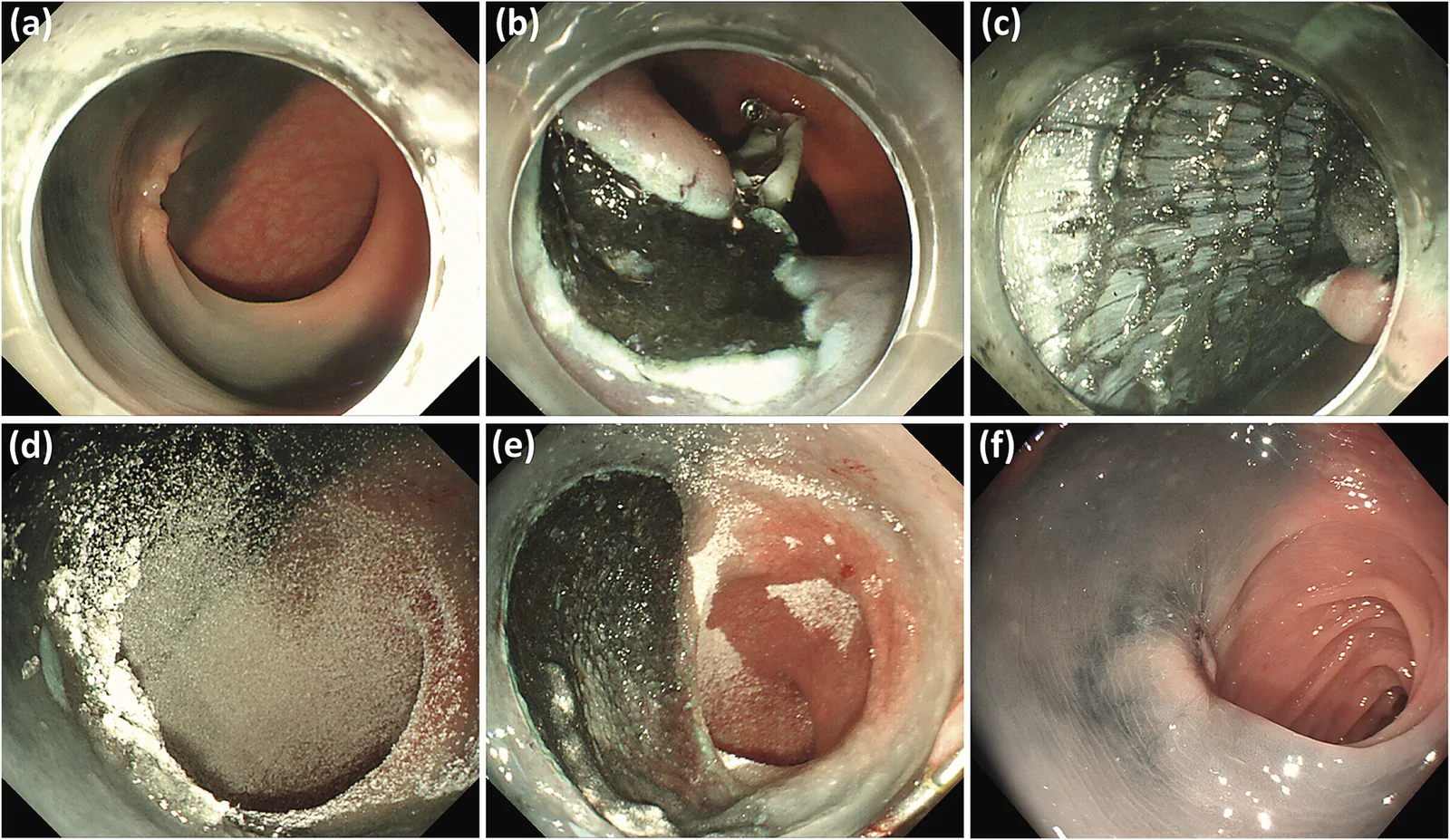
Traction-assisted ESD technique and hemostatic powder application. ( **a** ) Pre-operative view showing the target lesion. ( **b** ) Double-clip rubber band traction system providing effective countertraction. ( **c** ) Submucosal dissection adjacent to the muscularis propria using a small point incision technique. ( **d** ) Application of novel hemostatic powder (EndoClot) to the mucosal defect. ( **e** ) Water instillation to form an adhesive gel layer over the wound bed. ( **f** ) Follow-up colonoscopy at 25 days post-ESD demonstrating nearly complete mucosal healing.

A 63-year-old man underwent endoscopic mucosal resection (EMR) of a colonic lesion, with histopathology revealing adenocarcinoma ( Fig. 1a ). Carbon nanoparticle tattooing was performed for planned laparoscopic surgery, but the procedure was aborted due to pneumoperitoneum intolerance. Salvage ESD was subsequently performed.

During ESD, carbon nanoparticle tattooing caused prominent submucosal black discoloration and only localized central fibrosis. Despite excellent peripheral lift, the intense staining severely obscured the dissection plane, making layer identification extremely difficult. Following circumferential incision, a double-clip rubber band traction system was applied to provide stable countertraction and excellent exposure of the discolored submucosa 3 ( Fig. 1b ). Submucosal dissection was performed close to the muscularis propria using a small point incision technique ( Fig. 1c ). Intraoperative hemostasis was optimized using underwater endoscopic visualization. 4

The mucosal defect was treated with a novel hemostatic powder (EndoClot), which was transformed by gentle water instillation into a tight adhesive gel layer intimately conforming to the wound bed ( Fig. 1d,e ). 5 For this large defect, clip closure would have required numerous clips and likely achieved only superficial approximation; instead, the powder provided uniform coverage and durable protection, isolating the wound from intestinal contents while promoting healing.

Histopathology confirmed R0 resection. Follow-up colonoscopy at 25 days demonstrated nearly complete mucosal healing with no residual lesion or delayed bleeding ( Fig. 1f ). This outcome highlights the advantage of hemostatic powder in promoting physiologic healing while avoiding clip-related complications. This integrated strategy offers a new and effective option for lesions with an obscured submucosa layer and a high risk of adverse events.
